# New Perspectives in Overcoming Bulk-Fill Composite Polymerization Shrinkage: The Impact of Curing Mode and Layering

**DOI:** 10.3390/dj12060171

**Published:** 2024-06-05

**Authors:** Zsuzsanna Bardocz-Veres, Mátyás Levente Miklós, Edina-Kata Biró, Éva Andrea Kántor, József Kántor, Csaba Dudás, Bernadette Kerekes-Máthé

**Affiliations:** 1 Faculty of Dental Medicine, George Emil Palade University of Medicine, Pharmacy, Science and Technology of Târgu Mureș, 38 Gh. Marinescu Str., 540139 Târgu Mureș, Romania; zsuzsanna.bardocz-veres@umfst.ro (Z.B.-V.);; 2 Department of Mechanical Engineering, Faculty of Technical and Human Sciences, Sapientia Hungarian University of Transylvania, Târgu-Mureş, Târgu-Mureş/Corunca, Calea Sighișoarei nr. 2., 540485 Târgu-Mureş, Romaniakantorjozsef@ms.sapientia.ro (J.K.)

**Keywords:** bulk-fill composite, polymerization shrinkage, cuspal deflection, light curing mode, layering techniques

## Abstract

Background: The purpose of this study was to investigate the effect of different light curing modes on the polymerization shrinkage of a bulk-fill composite and to evaluate the impact of two layering techniques on the cuspal deflection. Methods: Nine different light curing modes were tested on bulk-fill composite samples in aluminum MOD cavities. Intensity, duration, and illumination distance were the factors that changed during the different curing modes. The digital image correlation method was used to visually represent the displacement of carbon particles on the materials’ surface caused by shrinkage along both the horizontal and vertical axes. For simulating cuspal deflection, a separate protocol was used, with a bulk and horizontal layering technique. Results: The results showed that the largest horizontal displacements were present in the soft start group (6.00 ± 0.82 µm) and in the X-tra power group (5.67 ± 1.21 µm). The smallest horizontal displacements were detected in normal curing modes (4.00 ± 1.58 µm; 4.00 ± 2.68 µm). The largest vertical displacements, at the bottom layer, were present in the normal curing mode group with a 20 s curing time (5.22 ± 1.56 µm), while the smallest vertical displacements were shown in the X-tra power group (2.89 ± 0.60 µm). The observed particle displacements showing the shrinkage of the composite were correlated with the curing mode. The bulk-fill group showed less cuspal deflection than the horizontal layering group did, but the difference was not statistically significant (*p* = 0.575). Conclusions: Within the limitations of this in vitro study, it can be concluded that lower intensities of curing lights (1200 mW/cm^2^) may perform better from the point of view of material shrinkage than high and extreme light intensities do. The pulse delay mode might be recommended in the case of bulk-fill materials. The number of layers did not significantly affect the cuspal deflection in the case of the studied composite.

## 1. Introduction

Composites can be categorized based on many factors, including the size and proportion of the filler particles, their viscosity, and the polymerization process. For the light-induced polymerization process, a photoinitiator is introduced into the mixture of monomers. The initiator is activated by blue light with a maximum wavelength of around 465 nm. The composite undergoes rapid internal hardening within seconds while being exposed to high-intensity light initiation. Although many processes that impact the final mechanical properties of composites persist after the curing process, the composites, once exposed to light, possess sufficient hardness to finalize the restoration and achieve a polished surface [1].

There are multiple factors influencing the polymerization degree and shrinkage of the composite. Among these are the placement of the light source, the duration of exposure to light, the ratio of the bonded to unbonded surface area (C-factor), and the curing modes offered by the light source. Over the years, several light curing modes have emerged. The continuous curing process involves the exposure of the composite resin to constant-intensity light for a predetermined duration. Soft-start curing modes involve gradually increasing light intensity, which may decrease polymerization stress and result in better marginal integrity of the restoration. The pulse delay curing technique involves applying short pulses of light to the restoration, followed by brief pauses for stress relief. Different curing units from different manufacturers may provide different curing modes with various light intensity possibilities. The effectiveness of polymerization ranges from 35% to 80% in general. In the case of an insufficient penetration of light into the bottom layers of the composite filling, insufficient activation of the photoinitiator occurs in those areas, potentially resulting in the inadequate hardening of the composite [2]. 

Composite restorations are often constructed using multiple layers and different layering techniques. There are various layering strategies such as horizontal, vertical, oblique, centripetal buildup, split-increment horizontal, or successive cusp buildup techniques [3]. Layering is necessary in the case of conventional composites due to the limited hardening depth of the composite, which sets the maximum layer thickness that can be applied. Generally, this limit is around 2 mm. Incremental layering enables dentists to achieve more exact contouring of the composite material, resulting in a superior marginal adaptation to the tooth structure. Furthermore, it is widely accepted in the field of dentistry that employing progressive layering techniques can effectively minimize polymerization shrinkage and hence mitigate the visible effects of stress. In addition, the application of multiple layers of composite material enhances the visual appeal of the final restoration [4]. Bulk-fill composites instead allow for applications in a layer measuring 4–5 mm. Depending on the polymerization shrinkage of the materials, different layering and polymerization techniques are employed. In the case of high-viscosity bulk-fill composites, it may be safer to use layering techniques instead of the bulk technique since polymerization shrinkage stress might be decreased this way [5,6]. 

Polymerization shrinkage is a phenomenon that occurs during polymerization, resulting in a volumetric reduction of 1–6% [7]. During this process, the molecules must get closer to each other to establish chemical bonds. Polymerization shrinkage generates internal stress, affecting the bonded surfaces of the enamel and dentin. These localized stresses may result in a reduction in bonding forces. When polymerization shrinkage occurs and stress between the tooth tissue and the composite appears, the forces can reach a maximum value of 13 MPa. The internal stresses and stresses that form at the margins of the restoration, over time, can lead to marginal leakage, secondary caries, marginal discoloration, crack formation in the tooth enamel, or potentially more extensive damage leading to cusp deflection and fracture [8]. Cusp deflection occurs when the composite’s polymerization shrinkage stress interacts with the cavity wall’s compliance. This varies depending on the measurement method, tooth type, and cavity size. Measuring the deflection of cusp tips is one method of testing the stresses resulting from polymerization shrinkage. This phenomenon is common in teeth restored with composite material and occurs when the cusps of a tooth change shape due to the shrinkage of the material used in dental restorations. This event can undermine the strength and stability of the tooth, resulting in sensitivity after a dental procedure, microleakage around the filling, and the development of secondary caries [9]. All of the above-mentioned issues are quite significant in clinical practice.

Although polymerization instructions are provided for each material, it is important to note that these instructions may not always cover all the curing modes offered by many light curing units. The objective of our study was to identify the most favorable curing mode and layering strategy for bulk-fill dental composites to reduce shrinkage stress and cuspal deflection. This is particularly important when considering the accurate implementation of the research approach in dental practice. The null hypothesis states that there is no difference in polymerization shrinkage between different light curing modes and layering techniques for bulk-fill composites.

## 2. Materials and Methods

### 2.1. Specimen Preparation 

**Figure 1:**
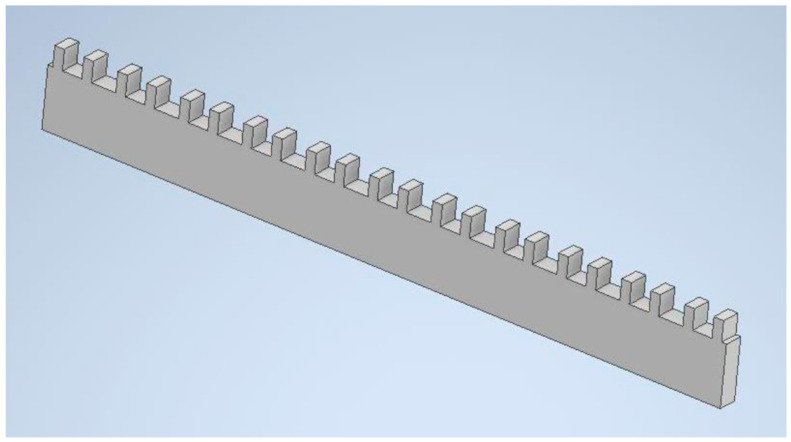
Three-dimensional model of the plate containing the cavities.

A 4 mm thick aluminum alloy (EN AW 2017, Amari Romania) sheet was used to fabricate the simulated MOD-type cavities. The 3D model of the component was created with cavities (Figure 1) using Autodesk Inventor Professional 2021 (Autodesk, Inc., San Francisco, CA, USA). Using this 3D model as a reference, the parts were manufactured on a CNC milling machine.

The cavities have dimensions of 4 mm × 4 mm × 4 mm, and the walls have a thickness of 2 mm on both sides. The inner surfaces of the cavities were sandblasted with 250 μm Al_2_O_3_ powder to improve retention. The air pressure used was 0.6 MPa. 

A dental adhesive, Prime&Bond NT (OptiBond Solo Plus, Kerr, Kerr Corporation, Bioggio, Switzerland), was applied in each cavity and completely covered all surfaces, in accordance with the manufacturer’s instructions, before each restoration. 

#### 2.1.1. Specimens for Different Curing Modes

The present in vitro study included a sample size of *n* = 5 per group, comprising a total sample size of 45. Therefore, a statistical power of 0.8 was used, and a significance level of 5% was applied using G*Power 3.1.9.7 software (Düsseldorf, Germany). In the first part of the present study, the cavities were filled using a bulk technique with Filtek One Bulk Fill A3 (3M ESPE AG, Bayern, Germany) composite. The inorganic portion of the material consisted of non-agglomerated silica and zirconia filler, aggregated zirconia/silica cluster filler, and agglomerated ytterbium trifluoride filler particles. The weight percentage of ytterbium trifluoride filler was 76.5%, while the volume percentage was 58.5%. The manufacturer’s recommended settings are 10–20 s with a minimum of a 800 mW/cm^2^ intensity curing unit in continuous curing mode, depending on the layer thickness.

All specimens were exposed to light emitted by a wireless LED curing unit (NOBLESSE, Max Dental Co., Bucheon, Korea) and cured for 3 to 20 s, using various light curing modes. The light intensities were measured with a radiometer (Woodpecker Led & Halogen Dental Curing Light Meter Power Tester, Woodpecker.co S.A., Krakowska, Poland). The groups created are shown in Table 1.

#### 2.1.2. Specimens for Different Layering Techniques

In the second part of the study, the same bulk-fill material was used and inserted into the cavities using two layering techniques: bulk insertion in one single layer, and horizontal layering technique in two layers with a thickness of 2 mm each. In total, 10 cavities (5 for each technique) were used in this part of the study, and were prepared as described above. All specimens were exposed to the same Noblesse curing unit for 20 s using the normal, continuous light curing mode in the case of bulk insertion and for 10 s for each layer using the normal, continuous light curing mode in the case of horizontal layering, as proposed by the manufacturer.

### 2.2. Measurement with Digital Image Correlation (DIC) 

The DIC approach necessitates the placement of a black-and-white pattern on the observed surface. Two different approaches were used in the present study to observe the movement of the pattern, which involved two sets of specimens [9]. In the first set, different curing parameters were used between the specimens, while in the second set, the layering techniques differed.

#### 2.2.1. DIC in Case of Specimens with Different Curing Modes

**Figure 2:**
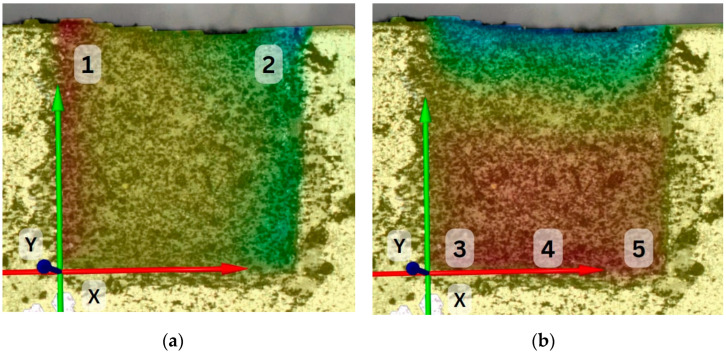
Examined points of the surface area in a PD-2x5-01 specimen along the horizontal (point number 1 and 2) (**a**) and vertical (point number 3, 4, and 5) (**b**) axes in lateral view.

After the placement of the composite material with the bulk technique in a layer measuring 4 mm, the lateral surface of the material was sprinkled with a thin layer of carbon dust, using the tip of a brush. The specimen was positioned at a 30 cm distance from a digital camera (Nikon D3100, Nikon Corporation, Tokyo, Japan, and Tamron 90mm macro lens, Tamron Co., Hasunuma, Japan). A remote control was used to operate the camera, to prevent any vibrations caused by direct handling. Before light initiation and right after that, a high-resolution photograph (6000 × 4000 pixels, ISO 100 sensitivity, 1/50 shutter speed, f22) was obtained. We enhanced the luminosity of the final photograph and altered its contrast using the Microsoft Photos (Microsoft Corporation, Redmond, Washington, DC, USA) application to facilitate the identification of the analyzed pattern by the DIC software (GOM Correlate, GOM Metrology, Leuven, Belgium). The images were correlated in the software, and the displacement of the particles was calculated. Each sample was examined at 5 different points of the surface area. Both horizontal and vertical particle movements were analyzed along the X and Y axes. Horizontal movements were analyzed at two points of the top layer, while vertical movements were analyzed at three points of the bottom layer (Figure 2). All the data were introduced into Excel (Microsoft Corporation) spreadsheets.

#### 2.2.2. DIC in Case of Specimens with Different Layering Techniques

In this part of the study, a very important step was to visualize the bending of the cavity walls, which simulated cuspal deflection. For this reason, a black-and-white pattern had to be realized on the surface of the cavity walls, too. A brush was used to administer two layers of white acrylic paint onto the sample surface. To obtain the intended texture, the painted surface was sprinkled with a thin layer of carbon dust, using the tip of a brush. The same imaging protocol as that in the previous description was used, with the difference being that in this case, images were taken before light initiation and 10 min after that. The photographs were taken from the occlusal view. The images were correlated in the GOM Correlate software (GOM Metrology, Leuven, Belgium), and the cuspal displacement was calculated by taking the sum of the absolute displacement values of the cavity walls on each side of the restoration.

### 2.3. Statistical Analysis 

Statistical analysis was performed in the SPSS software package (version 25.0, Chicago, IL, USA) using ANOVA and *t*-tests, conducting a comparison of the means of the various sample groups. The significance level was set at a *p*-value of <0.05.

## 3. Results

### 3.1. Results in Case of Specimens with Different Curing Modes

**Figure 3:**
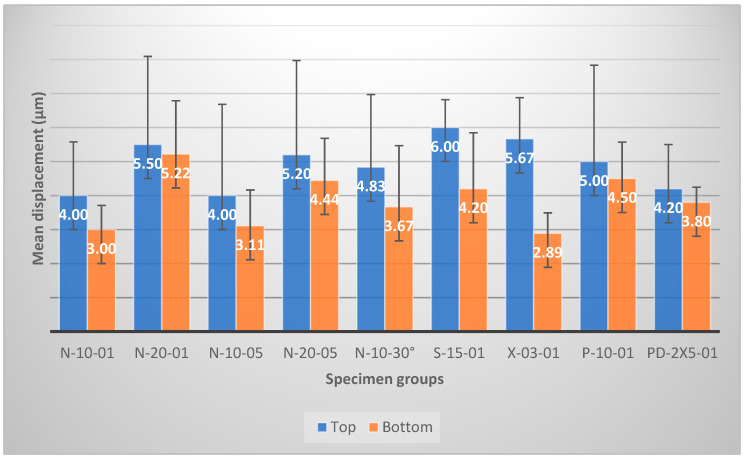
Mean values of particle displacement at the top and bottom layer of the restorations by group.

Mean values of the horizontal and vertical displacements along the X and Y axes are presented in Figure 3 for each specimen group. There were statistically no significant differences between the top (occlusal) and the bottom (pulpal) layers of the specimens (*p* > 0.05) regarding particle displacements only in the case of the soft start group (*p* = 0.04) and the X-tra power group (*p* = 0.00002). In both cases, the displacement values in the top layers were significantly higher than those in the bottom layers. The results showed that the largest horizontal displacements were present in the soft start group (S-15-01: 6.00 ± 0.82 µm) and in the X-tra power group (X-03-01: 5.67 ± 1.21 µm). The smallest horizontal displacements were detected in normal curing modes (N-10-01: 4.00 ± 1.58 µm; N-10-05: 4.00 ± 2.68 µm). The largest vertical displacements, on the bottom layer, were present in the normal curing mode group with a 20 s curing time (N-20-01: 5.22 ± 1.56 µm), while the smallest vertical displacements were shown in the X-tra power group (X-03-01: 2.89 ± 0.60 µm) and the normal groups with a normal curing time (N-10-01: 3.00 ± 0.71 µm; N-10-05: 3.11 ± 1.05 µm).

The observed particle displacements showing the shrinkage of the composite were correlated with the curing mode. The 10 s normal curing mode (*p* = 0.02) and the pulse delay curing mode (*p* = 0.02) resulted in a significantly lower level of horizontal displacement in the top layer compared with the soft start curing mode. The X-tra power mode resulted in a higher level of particle displacement than the normal mode for 10 s, with a 1 mm distance (*p* = 0.03). In the case of the normal, continuous groups from a 1 and 5 mm distance, statistically significant differences were detected only between the 20 s normal mode from 1 mm and the others (*p* = 0.01), regarding the vertical displacements. The same group showed significantly higher values than the X-tra power group (*p* = 0.0003) and the pulse delay group (*p* = 0.01) did. When comparing groups with 10 s of curing time (N-10-01, N-10-05, N-10-30°, and P-10-01), no significant difference was shown (*p* = 0.124). 

### 3.2. Results in the Case of Specimens with Different Layering Techniques

**Figure 4:**
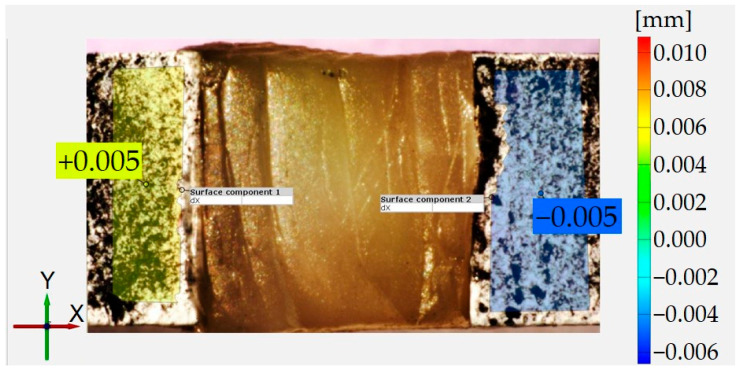
Cavity wall displacement in the horizontal (X) direction, as shown in the GOM correlate.

The correlated images showed that there were no displacement gradients along the cavity walls in any direction, and thus the displacement on either side of the restoration could be described with a single value (Figure 4). The displacement parallel to the walls was 0 μm in all cases, so only the perpendicular direction was considered.

The average total cuspal deflection values measured on five samples with DIC 10 min after the composite’s placement are shown in Table 2. The bulk-fill group showed less cuspal deflection than the horizontal layering group did, but analysis with the *t*-test indicated that the difference was not statistically significant (*p* = 0.575), so this may have been due to random chance. 

## 4. Discussion

A high-viscosity bulk-fill material was examined in the present study using the DIC method. Different light curing modes and two layering techniques were studied. The null hypothesis can be rejected due to the variability in the polymerization shrinkage behavior of the applied bulk-fill composite depending on the light curing mode and layering technique.

The DIC method allowed the observation of particle displacements at the interfaces between the cavity wall and the restoration. The displacements were observed both in the horizontal and in the vertical axes. During polymerization, composite molecules tend to move, causing the shrinkage of the material, which may generate stress, especially at the cavity wall/composite interface. This phenomenon was observed by several authors in the case of conventional and bulk-fill-type composites. Li et al. observed the vertical shrinkage of composite materials towards the bottom of the cavity and horizontal shrinkage towards the vertical midline of the cavity. Thus, the composite–cavity wall interface was highly affected, and cuspal deflections and high tensile strain were observed [10]. The results of the present study are in accordance with these findings.

The accuracy of DIC is influenced by the size of the speckles on the observation surface, as well as the subset window size. Several studies have determined that to minimize correlation error, the speckle size should be limited to a few pixels. Given the image resolution, the estimated size of the speckles is around 30 µm. This was accomplished with a thin layer of white paint and fine carbon powder, as previously explained in the second part of the study. The choice of an optimal subset window size in the present study was determined based on a reference, and several experimental attempts were conducted before selecting a size of 30 × 30 pixels. Increasing the size of subset windows can mitigate random mistakes by providing a greater number of patterns for picture matching. However, using larger subset windows has the disadvantage of sacrificing the inclusion of more complex information within them. Thus, provided the correlation error is within acceptable limits, it is always preferable to use a small window size, especially when dealing with extremely non-uniform displacement/strain maps and when focusing on local deformation [11,12].

In recent years, there have been significant advancements in LED curing lights. Some of the manufacturers offer different curing modes, and in some cases it might be difficult for the clinician to choose from these. Besides the different curing modes, the intensity and orientation of the light during the light initiation of a dental composite affect the shrinkage of the material. To avoid errors in the present study, the light curing unit was mounted on a stand to have a fixed position [13]. 

The amount of composite used in the specimens might be also a source of error. This was determined using an analytical balance to ensure the uniform distribution of the material throughout all the cavities analyzed. In the case of the two horizontal layer restorations, after each layer, the remaining material was stored in a light-proof box to ensure that the light in the room would not affect the results. Only artificial light was used to increase the durability of environmental effects in the experimental laboratory [14].

Several studies have shown that the pulse delay mode, a type of curing mode that includes a delay, provides a significant improvement in the marginal integrity of the restoration. In the case of the Noblesse curing light used in the present study, the pulse mode offered a 5 s interval of curing with a changeable intensity, and was completed with a 5 s delay. A study examined the efficacy of combining slow curing with no-intensity intervals between two initiations, one of a low intensity and the other of a high intensity. Yazici et al. showed that the use of the ramp and pulse delay light curing modes did not improve the marginal sealing of composite resins [15,16,17]. In the present study, the pulse-delay mode showed significantly lower values of particle displacements than normal curing modes and soft start curing modes did. 

The use of bulk-fill-type composite materials allows for a 4–5 mm layer thickness in one increment. Even so, there are studies dealing with the problem of layering in the case of high-viscosity bulk-fill materials to prevent the formation of polymerization shrinkage stress [5]. There has been a great deal of research into bulk-fill composites, and manufacturers have recently modified the composition of these materials in several ways. New generations of bulk-fill composites have been created with improved photoinitiator systems that allow for greater depth of cure. These composites also exhibit reduced volumetric shrinkage and shrinkage stress compared with earlier generations.

Assessing shrinkage and shrinkage stress during the polymerization of dental composites is a commonly used test method to assess the effect of the material on cavity walls. The shrinkage might be influenced by the dimensions and configuration of the cavity, as well as any structural defects of the tooth. For this reason, an aluminum alloy sheet of a substantial size was designed and created to simulate the cavities. The Young’s modulus of the aluminum alloy used (72.5 GPa) is very similar to the Young’s modulus of enamel. Therefore, it is suitable for accurately mimicking the deflection behavior of real tooth cusps. The dimensions of the cavities were chosen to closely resemble the actual size of MOD cavities that occur in teeth during the restoration process.

In the present study, no statistically significant differences were detected between the top and the bottom layers of the specimens (*p* > 0.05) regarding shrinkage in the case of the normal and pulse curing mode groups. In the case of the X-tra power group (*p* = 0.01), the particle displacement values in the top layers were significantly higher than those in the bottom layers. These results might have been related to the short curing time at a high intensity, and the bottom layers may not have received a sufficient intensity level of curing. A recent study investigated short curing times with high levels of intensity, and the researchers found that composites polymerized for 20 s showed a significantly increased degree of conversion where the samples polymerized for only 3 s but with a higher intensity [18]. 

However, Atria et al. showed that reducing light intensity and changing the protocol of the soft-start mode did not significantly change the polymerization shrinkage of the composite tested. They also observed the degrees of conversion after the polymerization process, but this was not affected by the different curing modes [19]. 

There were no statistically significant differences between the groups at 10 s of polymerization, in normal mode, using different distances and angles of the light curing unit tip from the composite surface. A possible explanation for this could be the similar composite mass movement with the same curing mode and time. During the pregel state of the composite, the mass movement caused by polymerization occurs as a result of particle movement. However, following the gel state, movement can occur due to internal stress or the stress at the margins. The movement of composite particles in the pregel phase is presumably affected by several factors, including the power output of the curing light, the distance and the angle of the tip of the curing light from the composite, as well as the focus of the curing light. We found no clear evidence of this in the present study. There is also a lack of comprehensive studies in the scientific literature referring to these factors. The few data that we found consider that the pregel phase and the particle movements of the composites usually take place in the first 5 s of light curing [20]. Further studies are required to gain a more profound understanding of these phenomena.

The layering method in the present study had no significant effect on the cuspal deflection. This suggests that the multi-layer application of bulk-fill dental composites does not lead to reduced shrinkage, and thus it is not necessary to work with a more complicated technique when using such materials. The light curing mode showed a greater impact on the shrinkage of the material than the layering technique did. This result is in concordance with that of other studies from the scientific literature [5,21].

Measuring the deflection of cuspal tips is one method of testing the stresses resulting from polymerization shrinkage. We discovered that the scientific literature primarily contains studies focusing on universal composites in correlation to cuspal deflection. Extensive research has been conducted on how the tips are affected by using different light curing materials and layering techniques to evaluate deflection. Kwon et al. found that cuspal deflection can be reduced using an incremental layering technique or a material with reduced polymerization shrinkage. They also used aluminum MOD cavities and found a reduction in deflection in the case of the incremental layering technique [22]. Park et al. studied cuspal deflection using a universal composite with several layering techniques. Their findings demonstrated that the bulk technique resulted in significantly greater deflection compared with the horizontal and oblique layering techniques [23]. Tsujimoto et al. conducted a study to investigate the effect of different layering strategies on cuspal deflection using bulk-fill composites. They used 4 mm × 8 mm × 4 mm aluminum MOD cavities in their work. Measurements showed a significant reduction in the deflection of simulated cuspal tips when incremental layering techniques were used for certain materials. However, other bulk materials showed no significant differences in deflection values between bulk and incremental layering techniques, similar to the findings of the present study [5].

Although the results of the present study are promising, limitations are a natural occurrence. The expertise of the operator can impact the quality of the restorations. The small sample size is also an important limitation. Additional research is necessary to evaluate factors influencing polymerization shrinkage by reproducing conditions observed in the oral cavity, as in vitro investigations do not accurately represent these settings.

## 5. Conclusions

Within the limitations of the present study, it can be concluded that lower intensities of curing light (1200 mW/cm^2^) may perform better from the point of view of material shrinkage than high and extreme light intensities can. The pulse delay mode might be recommended in the case of bulk-fill materials, but further studies need to be conducted to explore more pulse delay possibilities and variants. The number of layers did not affect significantly the cuspal deflection in the case of the studied composite, and the placement of the material should follow the manufacturer’s recommendations. According to the results, the clinical recommendation of the present study is that clinicians choose the bulk-fill composite to mitigate the adverse effects of restorations and streamline the filling process.

## Figures and Tables

**Table 1:** Groups of different curing protocols used in the present study.

Group	Curing Mode	Curing Time (sec)	Distance from Surface (mm)	Light Intensity of the Noblesse Curing Unit (mW/cm^2^)
N-10-01	Normal, continuous	10	1	1200
N-20-01	Normal, continuous	20	1	1200
N-10-05	Normal, continuous	10	5	1200
N-20-05	Normal, continuous	20	5	1200
N-10-30°	Normal, continuous	10	Inclined in 30°	1200
S-15-01	Soft Start	15	1	1400
X-03-01	X-tra Power	3	1	3000
P-10-01	Pulse	10	1	1800
PD-2x5-01	Pulse delay	Two times for 5 s, 5 s pause between	1	1800

**Table 2:** Total cuspal deflection values in the case of various layering techniques.

Layering Technique	Mean Values and Standard Deviation of Cuspal Deflection (µm)
Bulk	8.83 ± 1.17
Horizontal	8.33 ± 1.15

## Data Availability

The data presented in this study are available on request from the corresponding author.
